# Molecular Diagnosis of Helicobacter Pylori Strain by 16S rDNA PCR Amplification and Direct Sequencing

**DOI:** 10.4172/2155-9821.1000105e

**Published:** 2011-10-19

**Authors:** Hirendra nath Banerjee, Monique Gramby, Zack Hawkins

**Affiliations:** Department of Biological Sciences, Elizabethcity State University under, The University of North Carolina, Elizabethcity, NC-27909, USA

**Keywords:** Pcr, DNA sequencing, BLAST, *H.pylori*

## Abstract

**Aim:**

Rapid detection of H.pylori strains by PCR-Sequencing.

**Methods:**

16S rDNA amplification by PCR from template genomic DNA, confirmation of amplicon size by agarose gel electrophoresis, sequencing of amplicons by automated sequencer, analysis of sequences by NCBI –BLAST software.

**Results:**

The PCR –Sequencing and analysis of the sequence data by BLAST resulted in detection of the strain to be of H.pylori strain#26695.

**Conclusion:**

The pathogenicity of H.pylori depends on the strain of the bacteria, PCR-Sequencing and analysis of the sequence data by BLAST can be a very quick and useful diagnostic method of the pathogen.

## Introduction

There is an increasing demand worldwide for the application of intelligent, fast and inexpensive measurement systems in clinical diagnosis. In the field of Clinical Microbiology, current techniques generally require 24–48 hours to identify and characterize a pathogenic microorganism following a series of biochemical tests. Although new molecular biological and serological test have been introduced recently, they still have not replaced cultural methods and microscopy. Increased capital costs, need of highly skilled personnel and contamination, reduces the efficiency of these methods in the diagnosis of diseases like *H.pylori* infection and Tuberculosis [1,2]. Sequence analysis of the 16S ribosomal RNA gene has been raised as possible mean of bacterial identification, which may circumvent some of these difficulties [3–5].

The infection by *H.pylori* produces a superficial chronic gastritis, which can develop to Peptic Ulcer Disease, Gastric and Duodenal ulcer. In recent years it was found that *H.pylori* infection may play a role in the early stage of the sequence:atrophic gastritis, intestinal metaplasia, dysplasia, and gastric cancer. The relation between *H.pylori* infection and cancer is so close that in 1994, in the United States, the international Agency for Research in Cancer as well as the World Health Organization considered these bacteria as the most important causative agent of cancer clarifying it as a class 1 carcinogen.

Until now, several invasive methods have been used in the diagnosis of gastric infection produced by *H.pylori*: histology, culture, rapid Urea breath test but none of them are considered as a reference standard [6]. We present in this study a molecular approach for *H.pylori* detection in which the primary identification of the causative agent was accomplished by using broad range primers to amplify part of the 16S rDNA gene followed by sequencing of the amplicon and database search.

The routine clinical use of this technology appears feasible and this procedure could be used as a prototype for nucleic acid based amplification method for *H.pylori* detection.

## Material and Methods

Genomic DNA of two strains of *H.pylori*, Strain ATCC # 700824D and 70039D were purchased from American type culture collection (ATCC), Manassas, VA, USA. 16S rDNA was amplified with the universal bacterial primers [5];

Forward: 5′ AGA GTT TGA TCC TGG CTC AG3′
Reverse: 5′ AAG GAG GTG ATC CAG CCG CA3′

PCR reactions included 2.5 mMol/L dNTPs, 0.75 mol/L MgCl2, 1nMol/L primers (Forward and Reverse) and 2.5 units Taq in PCR buffer (Gibco/BRL, Gaithersburg, MD, USA). PCR tubes were heated at 94°C for 45s, 59°C for 60 s and 72°C for 90s in a Perkin Elmer DNA thermalcylcer. After 28 cycles, the primer extension period (72°C) was maintained for 7 min.

The DNA was sequenced by an automated DNA sequencer using the 5′ forward primer.

The resultant sequences were analyzed by NCBI-NIH BLAST search program for homology.

## Results

**Figure 1:**
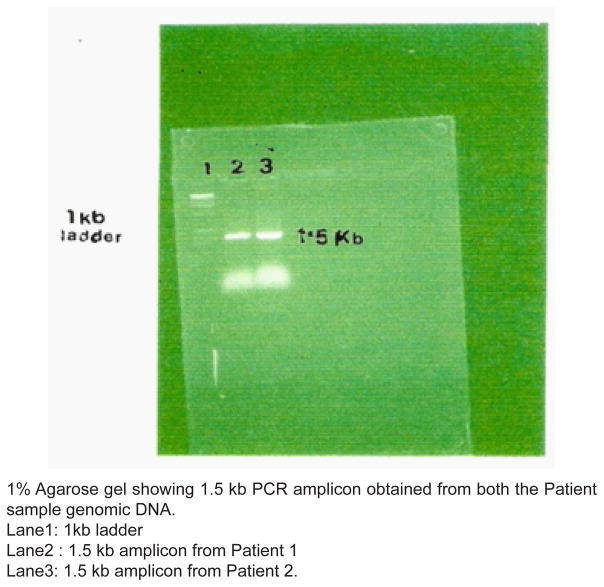
Gel-photograph.

A 1.5 kb amplification product was obtained from both the strains Figure 1. Partial sequencing of the amplicons was performed on an Applied Biosystems (373A) automatic sequencer using the 5′ Forward primer. The DNA sequences obtained were subjected to BLAST search (NCBI, USA) for sequence homology and showed high percentage of homology with 16S rDNA of *H.pylori* strain 26695. The sequences were reported to the GENBANK, and were accepted with annotation numbers: AY 057935 and AY057936.

## Discussion

PCR technology provides potential for a powerful diagnostic tool in detection of pathogenic microorganisms. A number of studies over the last ten years have employed broad range primers, such as those to the 16S rDNA genes, which can be used to detect most bacterial species. Similar with the conventional culture methodology, a molecular approach is as good as the specimen submitted and greater than the DNA database available. As of January 2000, the 16S rDNA sequences in the Ribosomal Database Project (RDP II) represents 2,460 different bacterial species [7–9]. In this study we have shown a rapid mean of identification and differentiation of two strains of *H.pylori* bacteria based on their 16S rDNA gene sequences. Since laboratory diagnosis of *H.pylori* by the existing methods has still less specificity and also it is difficult to diagnose re-infection, this method of detection by 16S rDNA could be a useful alternative. There are several potential advantages to 16S rDNA, PCR and sequencing. The technique is extremely sensitive and can detect DNA from a single infectious agent. Results can be obtained rapidly, unlike culture, which takes days to weeks. Therefore this technique is useful in clinical situations in which conventional microbiologic tests are too insensitive and slow or too cumbersome to be used on a large scale such as *H.pylori* detection. The pathogenicity of *H.pylori* depends on the strain of the bacteria, PCR-Sequencing and analysis of the sequence data by BLAST can be a very quick and useful diagnostic method of the pathogen
